# Supplementary figures and images for: NK cells modulate in vivo control of SARS-CoV-2 replication and suppression of lung damage
Source: PLoS Pathog. 2024 Aug 12;20(8):e1012439. doi: 10.1371/journal.ppat.1012439 (PMC11341101; doi:10.1371/journal.ppat.1012439)

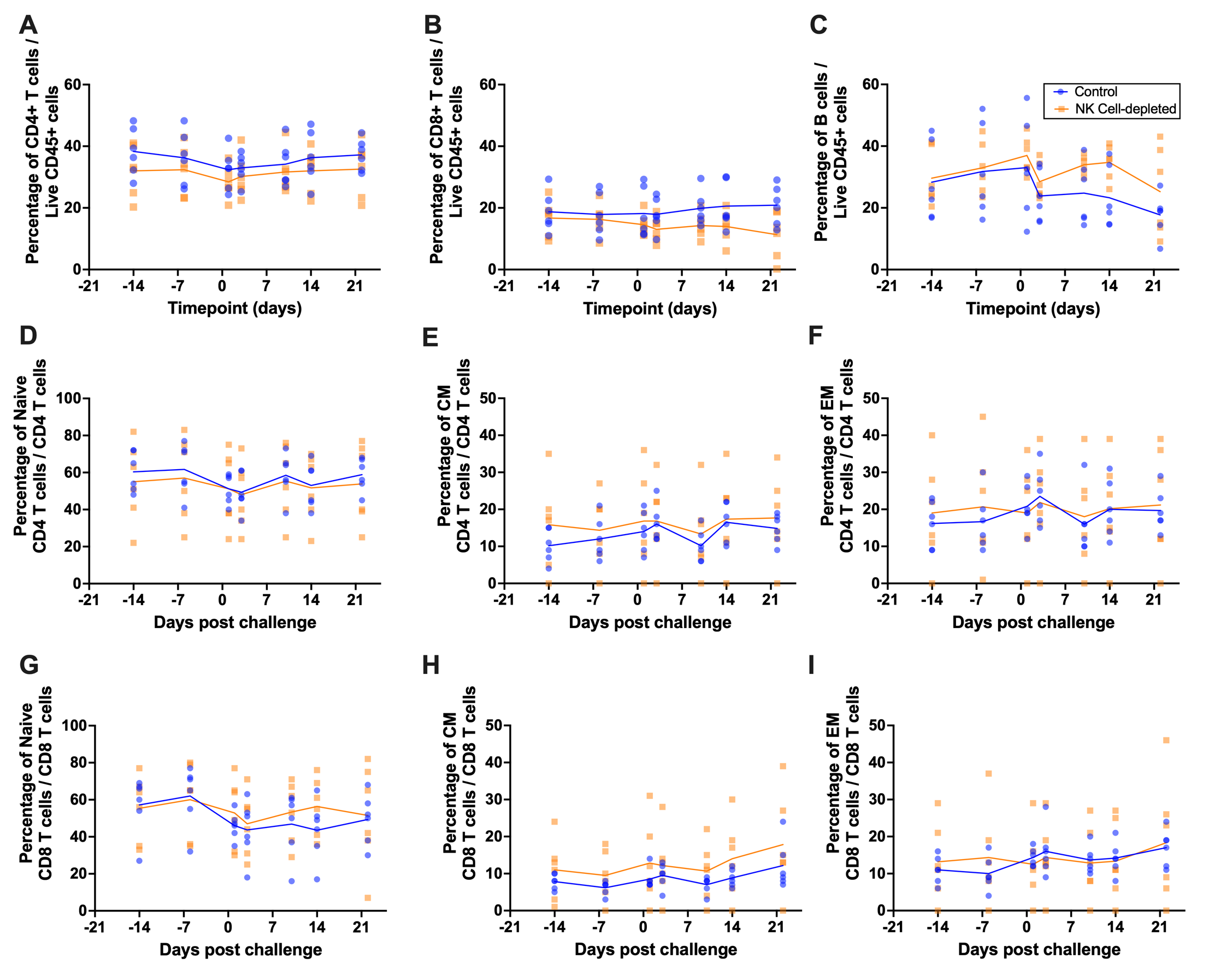

Supplement: S1 Fig — (A) Percent total CD4+ T cells; (B) Percent total CD8+ T cells; (C) Percent total B cells; (D) Percent naïve CD4+ T cells of total CD4+ T cells; (E) Percent central memory CD4+ T cells of total CD4+ T cells; (F) Percent effector memory CD4+ T cells of total CD4+ T cells; (G) Percent naïve CD8+ T cells of total CD8+ T cells; (H) Percent central memory CD8+ T cells of total CD8+ T cells; (I) Percent effector memory CD8+ T cells of total CD8+ T cells; Significance assessed by Two-way ANOVA. (TIFF) [file ppat.1012439.s001.tiff]

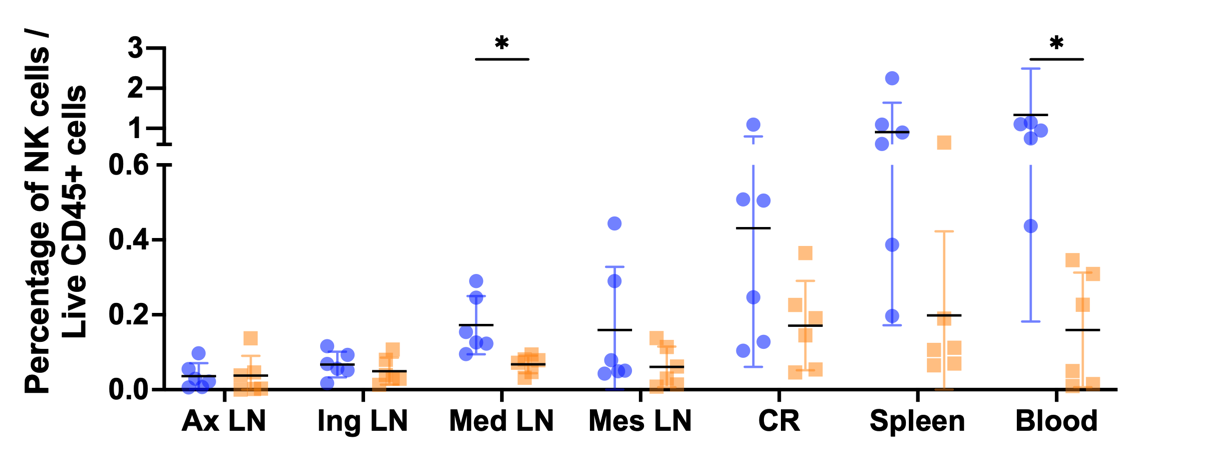

Supplement: S2 Fig — Blue–control group and Orange–NK cell-depleted. Black lines indicate mean and the self-colored lines indicate standard deviation. AX LN–Axillary lymph node; ING LN–Inguinal lymph node; Med LN–Mediastinal lymph node; Mes LN–Mesenteric lymph node; CR–Colorectal biopsy. Significance assessed by Multiple Mann Whitney tests; * adjusted p-value ≤ 0.05 (TIFF) [file ppat.1012439.s002.tiff]

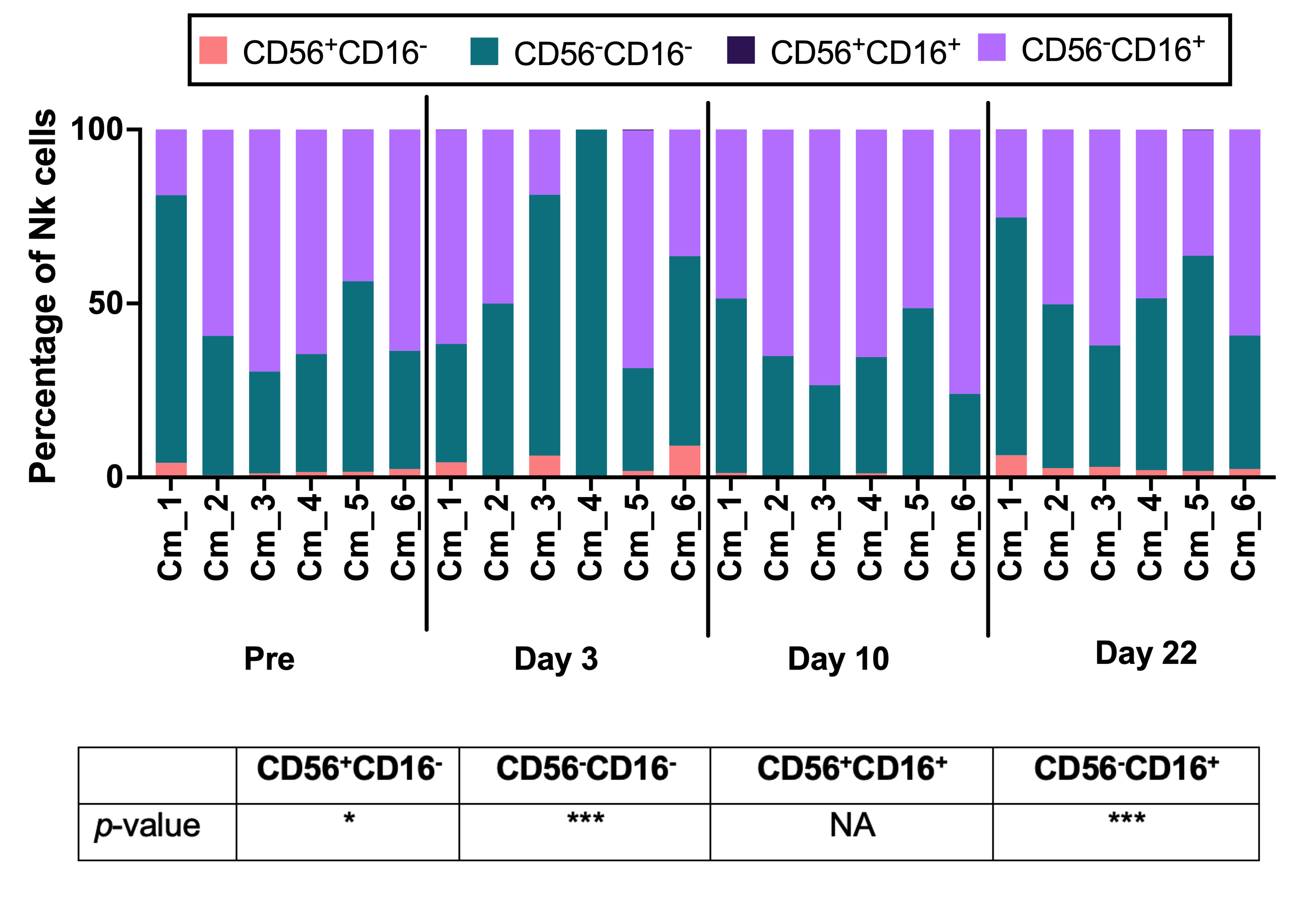

Supplement: S3 Fig — Table below main figure indicates the statistically significant differences in frequency from 10DPI to 22DPI. Significance assessed by One-Way ANOVA (* adjusted p-value ≤ 0.05; *** adjusted p-value ≤ 0.001). (TIFF) [file ppat.1012439.s003.tiff]

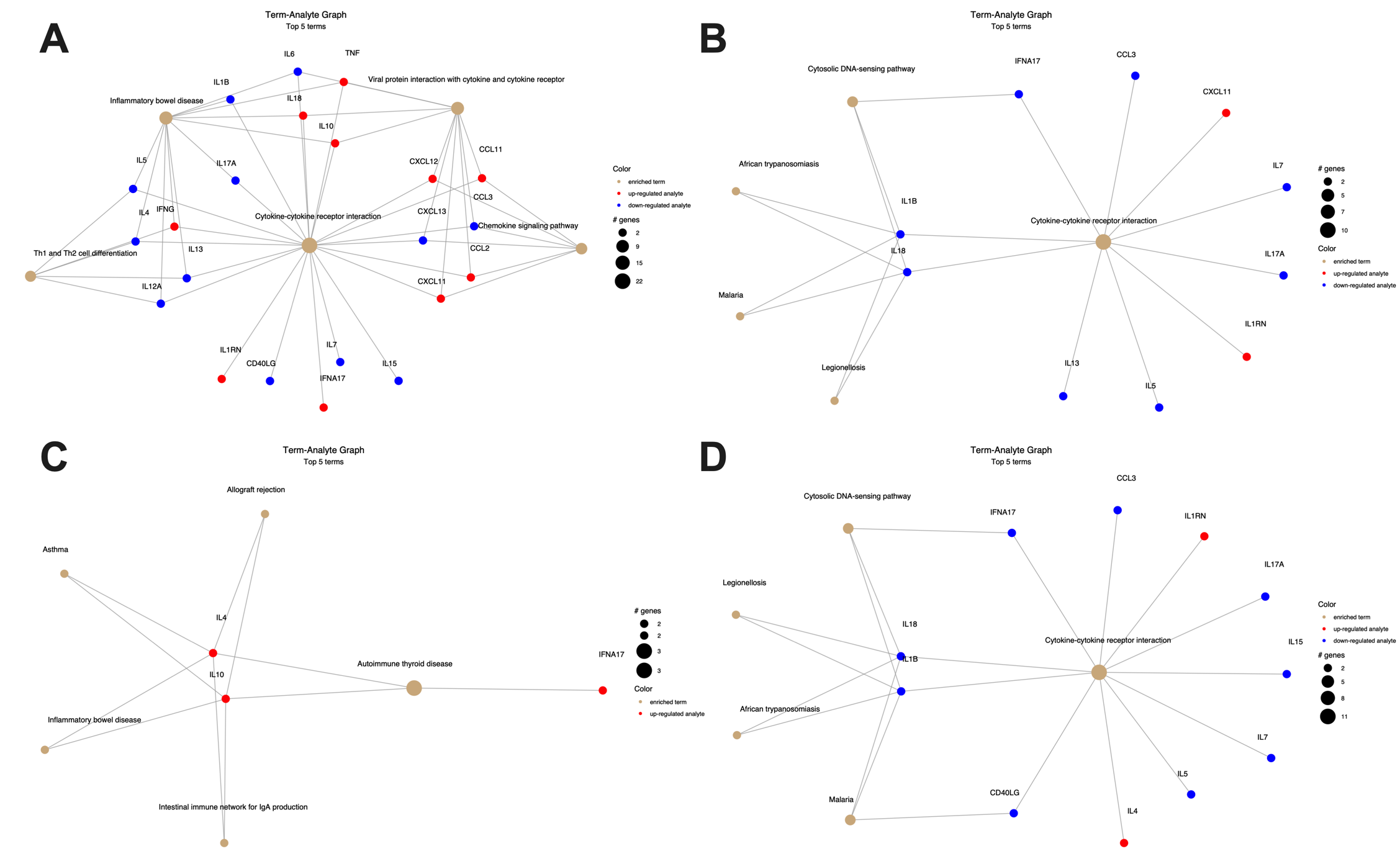

Supplement: S4 Fig — (A)–(B) Control group 3DPI and 14DPI; (C)–(D) NK cell-depleted group 3DPI and 14DPI. (TIFF) [file ppat.1012439.s004.tiff]

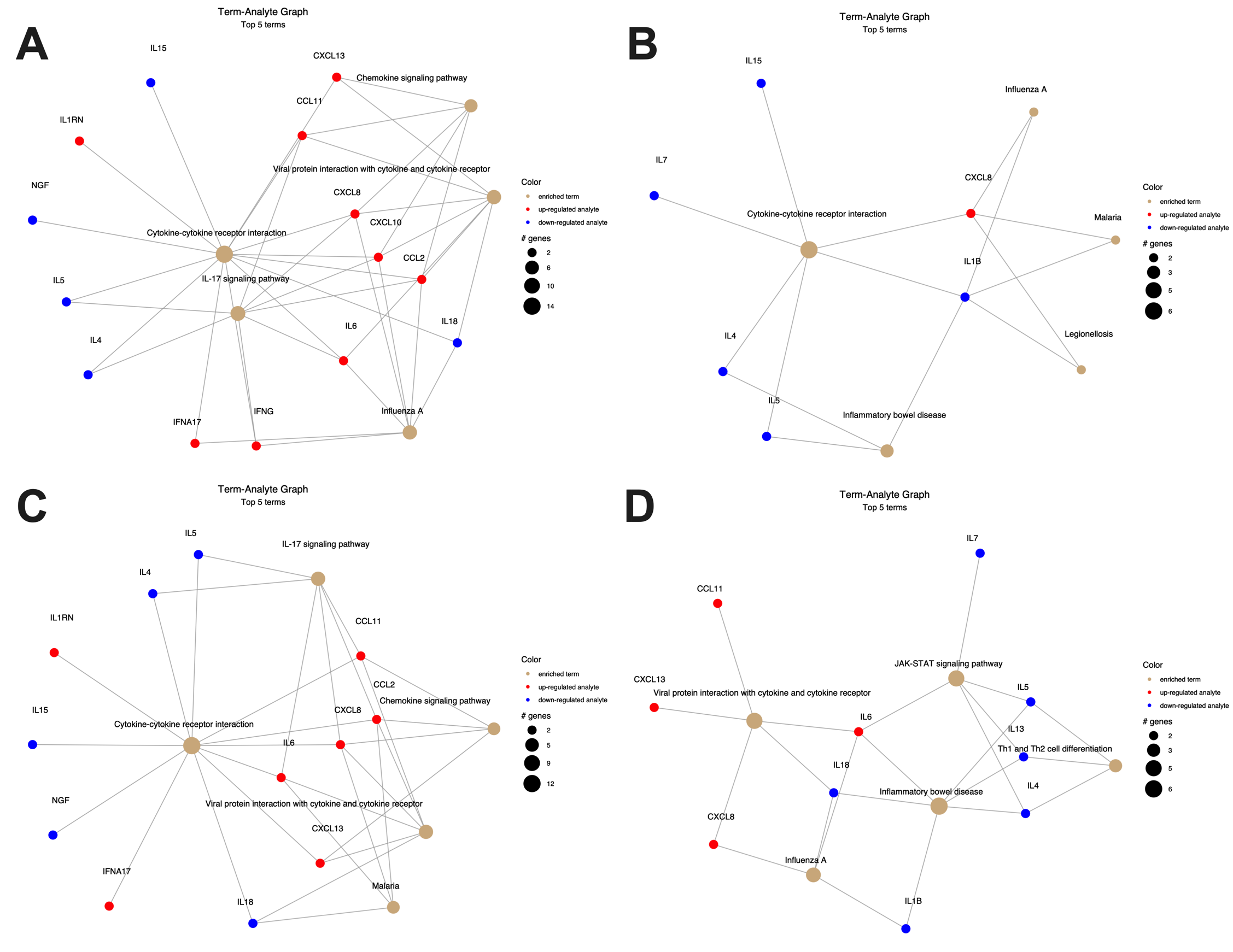

Supplement: S5 Fig — (A)–(B) Control group 3DPI and 10DPI (C)–(D) NK cell-depleted group 3DPI and 10DPI. (TIFF) [file ppat.1012439.s005.tiff]

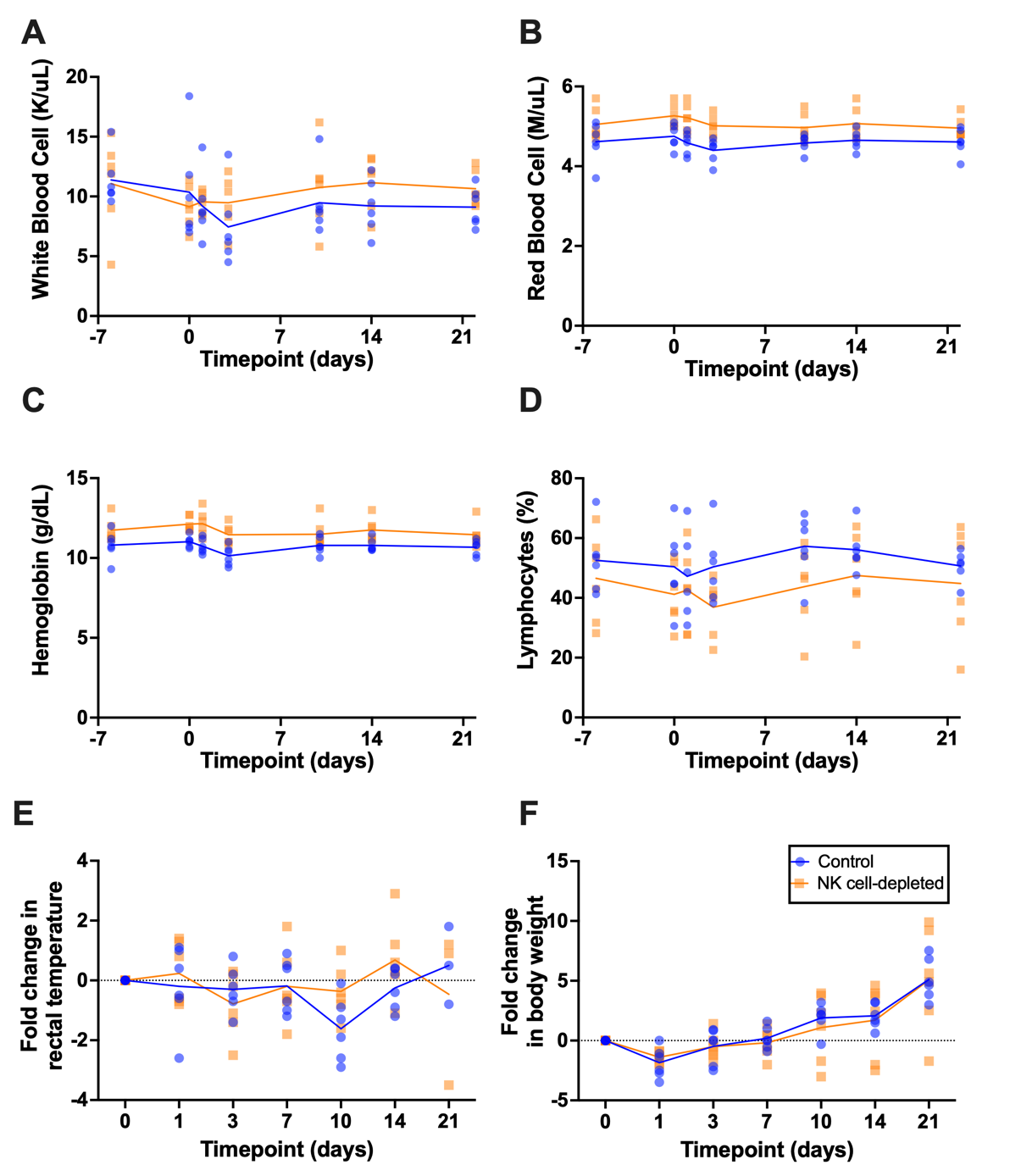

Supplement: S6 Fig — (A) White blood cells; (B) Red blood cells; (C) Hemoglobin; (D) Percentage of lymphocytes; (E) Fold change in body weights and (F) Fold change in rectal temperatures. K–thousand; M- million; g- gram. Significance assessed by Two-way ANOVA. (TIFF) [file ppat.1012439.s006.tiff]

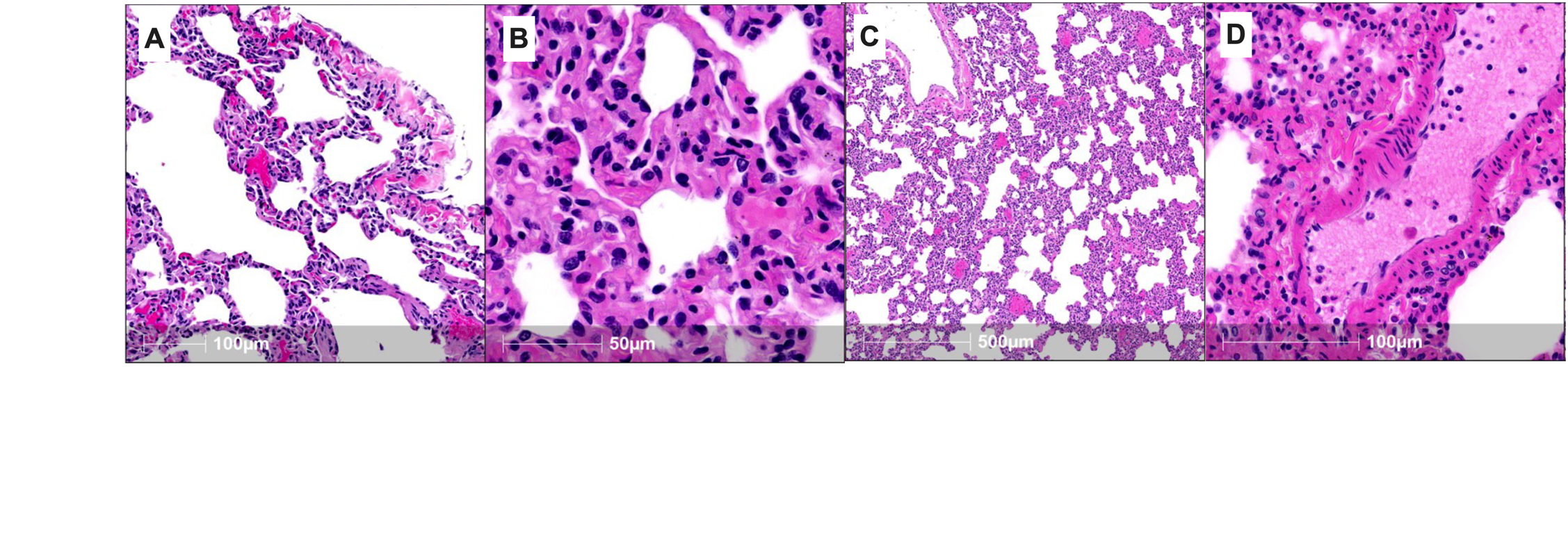

Supplement: S7 Fig — (A)—(D) H & E Staining on SARS-CoV-2 infected lungs of the control group indicating focal fibrosis, syncytia, type II pneumocyte hyperplasia, and endothelialitis respectively. (TIFF) [file ppat.1012439.s007.tiff]

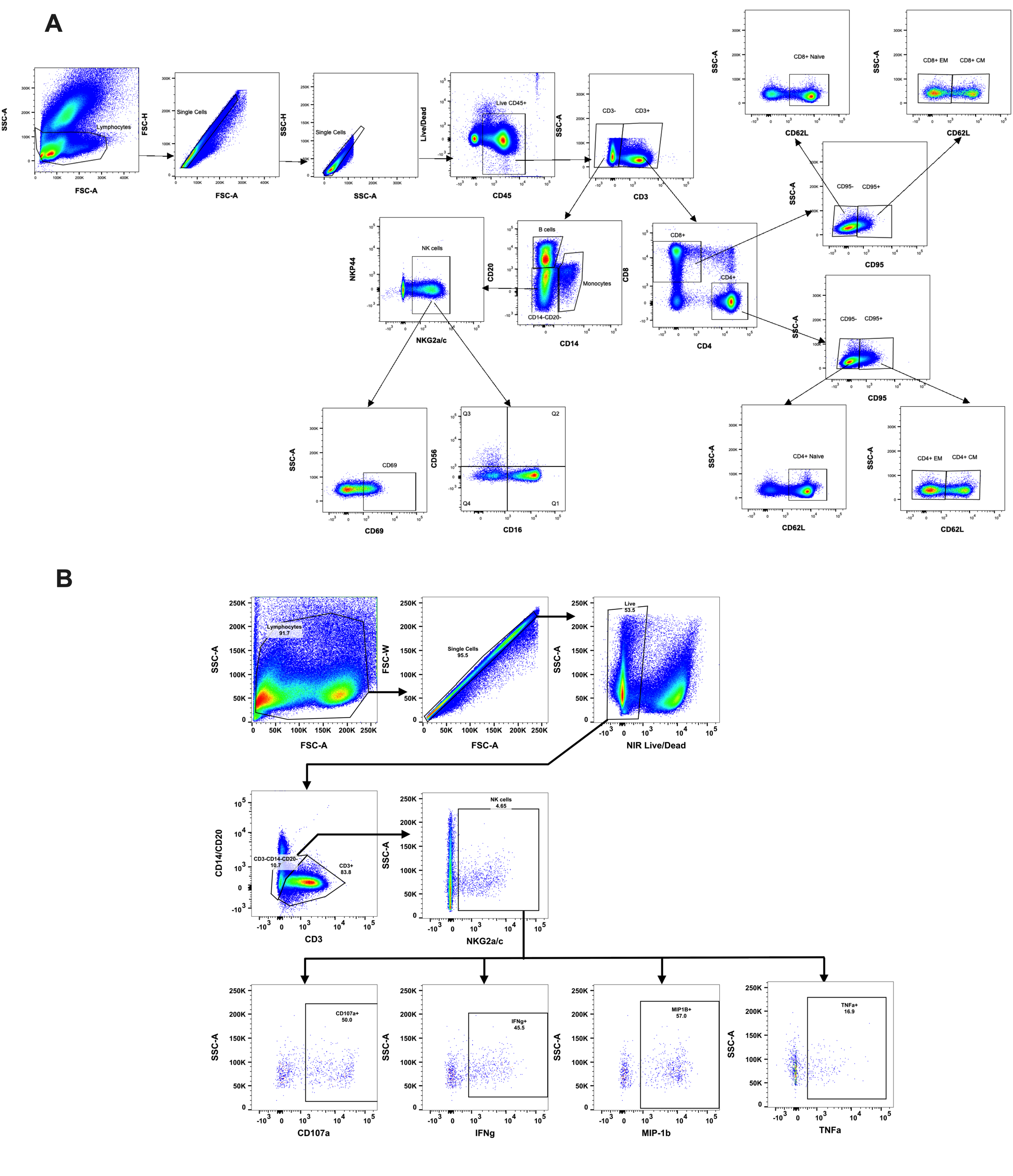

Supplement: S8 Fig — Flow cytometry gating strategies for (A) NK cell and T cell phenotyping; (B) Intracellular cytokine staining. (TIFF) [file ppat.1012439.s008.tiff]
