# Supplementary material for: NK cells modulate in vivo control of SARS-CoV-2 replication and suppression of lung damage
Source: PLoS Pathog. 2024 Aug 12;20(8):e1012439. doi: 10.1371/journal.ppat.1012439 (PMC11341101; doi:10.1371/journal.ppat.1012439)
Supplement: S1 Table — (DOCX) [file ppat.1012439.s009.docx]

| **Antibody-fluorophore** | **Clone** | **Company** | **Catalog number** |
| --- | --- | --- | --- |
| a4b7 APC |  | NHP resources | AB_2716326 |
| CD107a BV605 | H4A3 | Biolegend | 328633 |
| CD11b BV605 | ICRF44 | BD Pharmingen | 562721 |
| CD123 BV421 | 9F5 | BD Pharmingen | 565928 |
| CD14 BUV615 | M5E2 | BD Pharmingen | 751150 |
| CD159a PE-Cy7 | Z199 | BECKMAN COULTER | B10246 |
| CD16 BUV496 | 3G8 | BD Pharmingen | 612944 |
| CD195 (CCR5) | 3A9 | BD Pharmingen | 742913 |
| CD20 BV570 | 2H7 | BIOLEGEND | 302332 |
| CD20/CD14 BV570 | 2H7/M5E2 | BIOLEGEND | 302332/301832 |
| CD279 BV750 | EH12.1 | BD Pharmingen | 747446 |
| CD3 BUV395 | SP34.2 | BD Pharmingen | 564117 |
| CD335 (NKp46) PE-Cy5 | BAB281 | BECKMAN COULTER | IM3711 |
| CD336 (NKp44) PE | P44-8 | BIOLEGEND | 325107 |
| CD4 BB700 | L200 | BD Pharmingen | 566516 |
| CD45 BV786 | D058-1283 | BD Pharmingen | 563861 |
| CD56 BUV737 | B159 | BD Pharmingen | 741842 |
| CD62L BUV805 | SK11 | BD Pharmingen | 749209 |
| CD66abce BB515 | TET2 | MILTENYI | 130-116-522 |
| CD69 BV650 | FN50 | BIOLEGEND | 310934 |
| CD8 BUV563 | RPA-T8 | BD Pharmingen | 612914 |
| CD89 AF700 | A59 | Biolegend | 354117 |
| CD95 PE CF594 | DX2 | BD Pharmingen | 562395 |
| GZMB PE | GB11 | BD | 561142 |
| HLA-DR | G46-6 | BD Pharmingen | 612980 |
| IFNg BV711 | B27 | BD Pharmingen | 564039 |
| MIP-B AF647 | 24006 | Biotechne | IC271R |
| NIR live/dead | — | Thermofisher Scientific | L10119 |
| Perforin FITC | Pf344 | mAbTech | 3465-7 |
| TNFa-AF700 | Mab11 | BD Pharmingen | 557996 |

**Supplementary Table 1. Flow cytometry antibodies used for phenotyping and ICS.**
