# Supplementary material for: NK cells modulate in vivo control of SARS-CoV-2 replication and suppression of lung damage
Source: PLoS Pathog. 2024 Aug 12;20(8):e1012439. doi: 10.1371/journal.ppat.1012439 (PMC11341101; doi:10.1371/journal.ppat.1012439)
Supplement: Attachment — Submitted filename: Reviewer Rebuttal v1.docx [file ppat.1012439.s010.docx]

First off, we sincerely apologize to all the Reviewers. Many of the datapoints and error bars were fully correct in uploaded figures but for reasons unknown to us did not translate into the PLoS-generated composite. This was not alerted to our group until after the review period and we apologize for all the confusion this caused. We are embarrassed and frustrated for this waste of the Reviewers’ time. The full datasets and correct figures are now included. Ironically we feel correction of this problem will answer most of the Reviewers’ comments, and beyond that we have taken all comments seriously and addressed them in full in this new version.

Reviewer's Responses to Questions

**Part I – Summary**

*Reviewer 1: While this is a novel approach to directly assess the function of NK cells in SARS-CoV-2 infection, I have some concerns that their depletion strategy administered concurrently during acute SARS-CoV-2 infection may complicate some of their analysis presented. Furthermore, the statistical claims in the paper are lacking rigor, and results are overstated Some sections could also use some clarification and polishing. Furthermore, figures are missing formatting, error bars and P-values.*

We greatly appreciate the Reviewer’s assessment of the novelty of our work and understand their experimental concerns, many of which are due to publisher conversion issues, and we address in full comments below.

*Reviewer #2: Balachandran and coworkers provide an interesting interventional experiment in a nonhuman primate model to evaluate the role of NK cells in SARS-CoV2 pathogenesis. They nicely summarize the literature in humans with COVID-19 that has suggested positive or negative roles that NK cells could be playing in disease, making this an excellent question to pose. A comprehensive set of assays demonstrated: 1) control animals exhibited a rapid expansion of NK cells and transient expression of markers consistent NK activation, proliferation and possibly trafficking; and 2) NK cell depletion led to higher and more persisting levels of virus in BAL fluid and pharyngeal secretions; increased levels of pro-inflammatory cytokines in lungs; and more histological damage in lungs as assessed by blinded pathological review and scoring. The Reeves laboratory is among the leaders in the NK field and in developing NHP models to evaluate the role of NK cells in pathogenesis and vaccines. The study is well designed and rigorously conducted and provides new information that adds to our understanding of early immune responses during acute SARS-CoV2 infection. The finding that pathological outcomes and proinflammatory cytokine signaling pathways are enhanced following NK depletion is especially interesting in suggesting that this damage resulted from more persistent exposure to virus or dysregulatory effects on inflammatory responses.*

Again, we are humbled by the very kind words of the Reviewer and recognition of the importance of our work.

*Reviewer #3: In this manuscript by Balachandran et al, the authors use a cynomolgus macaque model of NK cell depletion (via sustained IL-15 depletion) prior to SARS-CoV-2 infection (delta variant) and find that viral titers are somewhat higher on day 10 post infection in depleted animals than they are in control animals. These data suggest an important role for NK cells in viral clearance. Additionally, the study provides a comprehensive assessment of NK responses to this virus in an important and translational model. The study is well done and the manuscript is well-written. There are caveats to the approach that could impact interpretation of the data that need to be addressed.*

We genuinely appreciate the positive comments by Reviewer 3 and address all their minor concerns below in full.

**Part II – Major Issues: Key Experiments Required for Acceptance**

*Reviewer #1: Major Comment 1: Anti-IL15 mAb treatment may stimulate inflammatory responses, potentially complicating analysis on differences in cytokine levels during SARS-CoV-2 infection in NK cell depleted vs control groups.*

*a. Why was anti-IL15 administered concurrently during the SARS-CoV-2 challenge (1 and 7 DPI) when it could potentially complicate analysis? Reactions to anti-IL15 or homeostatic changes in other cell populations could further contribute to increased pro-inflammatory cytokine levels. Given the dosing strategy, it is unclear if the absence of NK cells during acute infection or the just the anti-IL15 administration itself could be causative of increased inflammatory cytokine levels observed.*

We can understand the concern raised by the Reviewer, but our previous depletion study in SIV infection of rhesus macaques (doi: [10.1128/jvi.01519-22](https://doi.org/10.1128%2Fjvi.01519-22)), as well as the study discussed here, indicate little to no impact of anti-IL-15 dosage on the homeostasis of other immune cells (**Fig. 1C, Supplementary Fig 1**). To ensure sustained NK cell depletion, weekly doses of anti-IL15 administration are essential, hence the multi-dose strategy employed here. Day 1DPI was selected instead of 0DPI so that no other drugs would be administered to the cynomolgus macaques (CM) during the day of infection. Considering that the anti-IL-15 administration also happens at 7DPI and the cytokine levels in the BAL returned to the baseline levels at 10DPI, we are confident that the increased inflammatory cytokine levels observed at 3DPI are due to the absence of NK cells and not an off-target effect of the anti-IL15 dosing.

*b. It appears samples were collected at -6/7 DPI (post depletion but pre infection), were there comparisons done between depleted/control group in cytokine levels at this timepoint? If there wasn’t significant difference in cytokine profiles between depletion/control groups at this timepoint this could potentially validate the anti-IL15 dosage regime did not complicated analysis.*

We thank the Reviewer for this great suggestion. We performed additional cytokine analysis for the -6DPI timepoint and included our findings in **Fig. 3C**. Our observation was that there were no differences between the two groups at -6DPI (after the first dose of anti-IL-15 antibody), leading us to confidently state that the depletion strategy has not affected the cytokine analysis. This makes the impact of infection and lack of NK cells particularly evident for IFN-alpha and we highlight those findings in the revised paper.

*Major Comment 2: Statistical analysis of claims in paper are lacking rigor.*

*a. Statistical differences in viral loads are based on a selective analysis of a single time point (day 10), when differences in viral loads between the NK cell-depleted and the control groups were greatest (Fig. 2E, 2F, 2G & 2H).*

We performed statistical analysis on all the timepoints in the study. We have reported only the significant differences in the graph as asterisks and reported the *p*-values in the manuscript. The only significant differences in viral load (VL) between the groups were observed at 10DPI. Our decision to highlight this was due to the observation that NK cell frequency in the control group peaked on 10DPI as well, but felt it important to include all data for full study transparency.

*b. Statistical differences in viral load clearance from BAL and pharynx are not believable. With six animals per group and a difference in viral clearance at a single time point, it is difficult to believe that differences in the rate of viral clearance all reach a significance of p<0.0001 (Fig. 2I, 2J, 2K & 2L). This is particularly true for Fig. 2L, where clearance rates for the NK cell-depleted and control groups nearly overlap. The raw data needs to be provided to independently verify these claims.*

We thank the Reviewer for alerting us to the discrepancy. The statistics performed initially had taken into consideration all the survival curves across all anatomical compartments and VL measurements and compared them based on the group. We restricted our statistical reanalysis to individual compartments and individual VL measurements and have modified the graphs and the manuscript to reflect that the survival curves were statistically different between the groups only in the BAL compartment and that there was a reduced *p*-value based on the recommended Log-rank (Mantel-Cox) test. The raw data indicating the day of viral clearance are also below.

|  |  | BAL Sub G (DPI) | BAL Total E (DPI) | Pharynx Sub G (DPI) | Pharynx Total E (DPI) |
| --- | --- | --- | --- | --- | --- |
| Control | 1 | 14 | 14 | 14 | 14 |
|  | 2 | 14 | 14 | 14 | 14 |
|  | 3 | 14 | 14 | 14 | 14 |
|  | 4 | 10 | 10 | 10 | 10 |
|  | 5 | 14 | 22 | 14 | 22 |
|  | 6 | 14 | 14 | 14 | 14 |
| Depleted | 1 | 14 | 22 | 22 | 22 |
|  | 2 | 22 | 22 | 14 | 10 |
|  | 3 | 14 | 22 | 10 | 10 |
|  | 4 | 14 | 14 | 22 | 22 |
|  | 5 | 22 | 22 | 14 | 14 |
|  | 6 | 22 | 22 | 10 | 14 |

*c. While the manuscript claims there was no difference in T cells (bulk and CD4/CD8) between the depleted and control group, no statistical analysis was done to validate that claim. There is no statistical analysis presented for Figure 1C, and S1AB. Furthermore, it is not presented if there was any difference in B-cell homeostasis between the two groups.*

Apologies for the confusion. We did run statistics on the T cell comparing the depleted and control groups but there were no statistically significant differences. Throughout the manuscript, we have reported only the significant *p*-values and in the figures used asterisks to indicate the same. The B cell frequencies were also not statistically different between the groups throughout the observation window, although we have now added these values in **Supplementary Figure 1C**. The manuscript has been modified to include the name of the statistical test used in the corresponding figure legends.  *d. P-values and individual data points are missing from a number of the figures (Figures 1, 3, 4, 5, S1, S2, S3, S6). It appears some of these issues are fixed on the previous preprint in Research Square, but it is an oversight if these were not corrected in the submission.*

We are hoping that a majority of these issues identified by the reviewer will be fixed in this re-rendered manuscript. *P*-values are reported as asterisks in **Figures 1, 3, and 5**, and the values are reported in the text. For **Fig. 1E**, there are two CM that have very similar data points leading to almost complete overlap. (6.025 and 5.915 during the pre-timepoint and 10.64 and 10.55 at 22DPI). For **Supplementary Figure 2**, the asterisks have been added; for **Supplementary Figure 3** the *p*-values as asterisks are mentioned in the table below. The individual data points are all included.

*Major Comment 3: Given the relatively minor differences in viral loads between the experimental and control groups and the selective statistical analysis of the viral load data, the title of the manuscript claiming that NK cells are critical for in vivo control of SARS-CoV-2 replication is grossly overstated and needs to be revised.*

We respectfully would like to bring to the attention of the Reviewer that the VL variations and the difference in the VL kinetics, despite being minor, are statistically significant. The statistically significant difference in the VL between the two groups occurring only at 10DPI, when there is peak NK cell frequency in the control group, is a major indicator of the importance of NK cells and a two-log change in virus shedding is not minor. Further, the impact of NK cell depletion on lung pathology and the massive induction of inflammatory IFN-a in BAL compared to the control group are not minor either. We take issue with the Reviewer’s false claim that we are overstating any data, but we have dialed back the title in the spirit of compromise.

*Reviewer #2: The only major point to note is that the authors should say more about what the NHP model(s) have been able to show for SARS-CoV2 infection and what their limitations have been. This model is excellent for demonstrating transient infection, but not for the recapitulating the acute fulminant pathology of severe COVID-19 in humans. Just a few comments for a general audience with references on the state-of-the art for this NHP model would provide a useful perspective while not mitigating their observations.*

We appreciate the Reviewers’ comment and have added additional text to the manuscript.

Non-human primate (NHP) models are pertinent to infectious disease research due to their physiological, genetic, and immunological similarities to humans.^85^ The ACE2 receptors binding to SARS-CoV-2 are identical between NHP and humans, whereas mouse ACE2 binding is limited.^86,87^ We acknowledge that the NHP model in our study demonstrates transient infection and not the acute fulminant pathology of severe COVID-19 in humans and fails to address disease features like ARDS, coagulopathy, systemic sequelae, and mortality.^88^ But since SARS-CoV-2 infected NHP develop mild to moderate respiratory disease followed by full recovery our model recapitulates the infection in humans.^89^

***Part III – Minor Issues: Editorial and Data Presentation Modifications*** *Please use this section for editorial suggestions as well as relatively minor modifications of existing data that would enhance clarity.*

*Reviewer #1: Minor comment 1: The cytokine network analysis is somewhat lacking and poorly worded (line 225-237). Furthermore, no description of the methodology is included in methods section.*

We apologize for the lack of clarity. This wording has been modified and the Methods section has also been updated. For the cytokine networking analysis, Luminex cytokine data were loaded into R v4.3. Samples were grouped by study group and timepoint, and means for each cytokine were taken. Fold change values were then calculated for each group and timepoint by dividing each timepoint by the pre-infection timepoint and then log2 was transformed. Cytokine names, log2 fold change values, and *p*-values were assembled into a data frame and as input for the standard pathfinder workflow utilizing the KEGG database for interaction network analysis. Plots were generated with the term_gene_graph() function run on the result of pathfindR analysis.

*a. Does the analysis break down what networks are involved in “Viral protein interaction with cytokine and cytokine receptor”? I understand this term is enriched only at 3DPI for the control group whereas it was enriched at 3 DPI and 10 DPI for NK cell depleted, potentially consequence of the prolonged viremia, however, the explanation is poorly worded.*

We understand that our original explanation may have been confusing, and have now added language to explain the cytokines involved and the explanation for why this may be relevant. *b. Furthermore, it is referenced that complexity of the networks differs between timepoints, but it is not explained what relevance or conclusions could be drawn from this difference.*

We appreciate the Reviewer for pointing this out, we have added a line to explain the key takeaway for this statement.

*Minor Comment 2: Minor grammar/wording comments outlined below:
a. Line 76 “… (for example, NKG2D and NKP80)” for example is redundant, can be deleted.*

We have deleted this as the Reviewer suggested.

*b. Line 74-79 is a run-on sentence and confusingly written. Please reword.*

We thank the Reviewer for their comment. The sentence has been revised as follows:

“The virus-infected cells upregulate self-encoded molecules induced by the infection and/or cellular stress response (MHC class I-related chains (MIC) A and B and members of the UL16-binding protein (ULBP)) as well as pathogen-derived molecules. NK cells mediate the killing via the engagement of these molecules with the natural cytotoxicity receptors (NKp30, NKp44, and NKp46) and C-type lectin-like receptors (NKG2D and NKp80) on their surface.”

*c. Line 96,97 “NK cells in humans decline to 55%...” Believe it should be “declined by 55%”?*

This line has now been edited to say:

*“*The circulating levels of NK cells in humans declined to 55% during acute infection compared to pre-infection, which can be attributed to putative over-recruitment and homing of NK cells into lungs due to SARS-CoV-2-induced cytokine storm.”

*d. Line 124 in section title – Should be SARS-CoV-2 instead of SARSCoV2s to be consistent.*

We apologize for this inconsistency and have corrected as mentioned by the Reviewer.

*e. Figure 1A references a -6 DPI timepoint when others reference -7 DPI. Please correct/confirm*

**Fig. 1A** was in reference to the timeline of the study. The samples were indeed collected on -6DPI, but the graph’s the axis is in decimal form with a 7 day interval. With the correct rendering of the figure images during this round of submission, we hope that this timeline is now clearly represented.

*f. Line 268 “our control showed expanded NK…” should be our control group showed.*

We apologize for this error and have corrected as mentioned by the Reviewer.

*g. Line 280 “… role for NK cells in SARS-CoV-2, come from…” comma is unnecessary.*

We apologize for this error and have corrected as mentioned by the Reviewer.

*Reviewer #2: 1. In the abstract and at several points in the paper the term “viremia” is used. Since SARS-CoV2 is being measured in sections and not blood (as was the case in their JVirol paper on the role of NK cells in SIV infection), this term should not be used. Shedding would seem to be more appropriate.*

We thank the Reviewer for their suggestion. Changes have been made throughout the manuscript to indicate viral load or viral shedding distinctly.

*2. Line 160: describing human populations of NK cells in peripheral blood, they should refer to the “CD56brightCD16- cytokine-secreting…” cells.*

Apologies. The manuscript has been updated accordingly.

*3. Supp Fig 3 did not reproduce well in the version of the manuscript that came across on line. It looks like the margins of the bars were deleted. Only the CD56+CD16- and CD56-CD16- cell populations are shown in the bars.*

This issue was due to an error in the pdf file and has been rectified in this version.

*4. Line 190: For the general audience, add a descriptor or a line in the methods to indicate what subgenomic (Sub G) and total envelope (Total E) are actually measuring.*

We thank the Reviewer for their suggestion. We have modified the Methods section to read as:

“The total E measurements were included for SARS-CoV-2 infection monitoring while the subgenomic N gene mRNA is a potential measure of replicating virus and is used to differentiate productive infection from input virus.^93^”

*5. In Figure 3, Panels C and D, some data may not have transferred. Controls are not shown and the “Bar” on Day 3 is undefined.*

This was an error which occurred during the rendering of the document as a pdf. We have corrected this issue.

*6. In Fig 5, the histology panels B, C, D and E show only NK-depleted samples. It seems odd not to have any pictures of the Controls. Perhaps add more pictures in a supplemental figure? Also, the differences noted in histology producing the “Lung Lobe Score” were determined (Line 470) in a blinded manner “by a board-certified pathologist (A.J.M).” It’s not clear from the author list who this person is. If this is Amanda Martinot, please include her middle initial in the author list. If she was the pathologist, it would be worth noting that she was also the pathologist who apparently did the scoring in the cited Nature 2021 manuscript (Ref 59).*

Thank you for the valuable suggestion. We have added the images for the control group as **Supplementary Figure 7**. The initials for Dr. Amanda J. Martinot have been updated in the authors’ list.

*Reviewer #3: Overall, the manuscript could benefit from a more thorough description of how this study fits in the plethora of NHP SARS-CoV-2 studies. This is not the first study to use cynomolgus macaques with this virus nor the first to use the delta variant but it appears no other studies with this species or use of this variant in any NHP model are discussed or cited.*

We thank the Reviewer for their suggestion. We have updated the discussion to include these in addition to other citations requested from Reviewer 2:

“Airborne SARS-CoV-2 infection of CM has been reported to induce a model of COVID-19 more representative of human disease.^66^”

“A previous SARS-CoV-2 infection study (Washington strain 2019-nCoV/USA-WA1/2020) on RM using a T cell depletion strategy concluded that T cells are not critical for recovery from acute infection while B cells and antibodies play a significant role in the recovery process and immune memory.^30^ A CD8^+^ T cell depletion study using the same strain of virus as above indicated that CD8^+^ T cells may also contribute to protection when neutralizing antibody titers decline in an NHP model.^67^ Proteomic and metabolomic analyses of SARS-CoV-2 infected (BetaCoV/Beijing/IME-BJ05/2020) CMs have identified that the innate immune system, neutrophil and platelet activation and degranulation play central roles in mimicking a moderate COVID-19 disease.^68”^

*The use of the delta variant is an interesting and possibly important choice. Do the authors think the use of this virus, which showed somewhat increased virulence in humans and animal models, impacted their results? If so, this should be discussed.*

We thank the Reviewer for their comment and suggestion. Delta was used to best recapitulate a pathogenic infection with more virulence to truly get a sense of the role of NK cells. The Discussion section has been updated as follows:

“The Delta variant was selected in this study due to its phenotypic advantages, including early infection kinetics and enhanced induction of inflammatory biomarkers.^63^ Additionally, the Delta variant has a higher capacity to induce severe symptoms and an increased mortality rate in humans.^64,65”^

“One of the hallmarks of the Delta variant of SARS-CoV-2 is its enhanced ability to induce syncytia formation, which facilitates cell-to-cell viral spreading.^63^ This phenomenon was observed in both the groups in our study.”

*Importantly, a previous study was conducted with depletion of NK cells (as well as CD8 T cells) prior to infection using the anti CD8a antibody, and showed no effect on viral titer (Hasenkrug et al, DOI: 10.1128/mBio.01503-21). There were important differences in that study besides use of a different depletion technique (that study used rhesus macaques infected with the WA1 viral isolate). That manuscript should be cited and the different findings discussed.*

As suggested by the Reviewer, this relevant paper was mentioned in the Introduction section as such:

“In a T cell-depleted rhesus macaques (RM) model the delayed but efficient clearance of SARS-CoV-2 infection was orchestrated by virus-neutralizing antibodies.”

The paper was also discussed in the Discussion section:

“A previous SARS-CoV-2 infection study (Washington strain 2019-nCoV/USA-WA1/2020) on RM using a T cell depletion strategy concluded that T cells are not critical for recovery from acute SARS-CoV-2 infection while B cells and antibodies play a significant role in the recovery process and immune memory.^30^”

*The authors note that NK cell expansion peaked on day 10 in control animals, coincident with higher levels of viral RNA in the lung in depleted animals, but it appears many/most other markers of NK activity peak on day 3, coincident with the peak of viral titer in both groups of animals. This is worth mention.*

Thank you for the suggestion. As per the Reviewer’s suggestion the sentences have been modified to include the relevant information:

“Although peak VL were only marginally higher, at all subsequent timepoints post infection, the NK cell-depleted group had higher VL compared to the control group, reaching significance at 10DPI. Concurrent to peak VL at 3DPI we notice that the activation marker CD69 expression on NK cells in the control group increased, followed by an increase in the proliferation marker Ki67 expression at 10DPI which is when peak NK cell frequencies in this model are observed.”

*Previous studies have shown that IL-15 depletion does transiently deplete effector memory CD4 and 8 T cells and the authors cite one of these manuscripts (citation #50), but it’s not clear if the Tem cells were measured in this study? On a similar note, by day 10 when viral differences are noted between groups, adaptive immune responses are likely ramping up but no measures of either T cell or humoral immunity are shown. Are these data available? Given IL-15 depletion has a known (though subtle) impact on T cell immunity, is it possible the viral titer differences on day 10 are due to differences in T cell immunity (or another non-NK effect)?*

As the Reviewer mentioned, we were concerned about the off-target effects of anti-IL-15 depletion on the Tem subset. However, despite subtle changes in the NK cell-depleted group, no significant differences in this cell population were observed between the two groups. Graphs showing the different subsets of the T cells as well as bulk B cells have been included in the **Supplementary Figure 1C-I**. Additionally, we did not observe any dramatic increases in the adaptive immune responses in either group. No significant differences between the groups were observed in the bulk T cells, CD4/CD8 subsets, or the B cell levels throughout observation strongly indicating that the viral titer differences are affected by the NK cells (**Fig. 1C, Supplementary Figure 1**).  *I believe the word “viremia” is defined as describing virus in blood. This word is used throughout the manuscript to describe viral titer in other compartments. This should be changed.*

We thank the Reviewer for their suggestion. Changes have been made throughout the manuscript to indicate viral load or viral shedding distinctly.

*Supplemental Figure 1 suggests lower CD8 T cell numbers in NK depleted animals, particularly at later time points that are important for this study, but no statistics are shown. Is this difference significant?*

Two-way ANOVA test was the analysis used to assess significance when comparing the CD8 T cells between the groups. This information has been added to the figure legend. The adjusted *p*-value indicated that there were no significant differences between the two groups.

*Supplemental Figure 2 only shows black lines, not orange or blue as indicated in the legend.*

We thank the Reviewer for their comment. Again, this was a conversion issue. The revised manuscript has corrected figures.
